# Need for strategies to maximise student attainment at the clinical stage of training

**DOI:** 10.1080/10872981.2017.1364605

**Published:** 2017-08-17

**Authors:** Abdulla Bedir, Milad Golsharifi, Haleema Aya

**Affiliations:** ^a^ King’s College London, Guys Kings St Thomas’ School of Medicine, London, UK

We would like to thank Connor et al. immensely for their essential contribution ‘Improving clerkship preparedness: a hospital medicine elective for pre-clerkship students’ [1]. As senior medical students, we share the belief that there is a strong need to develop effective strategies to enhance student readiness for transition to the clinical phase of medical training.

It has certainly been our experience that opportunities to practise clinical skills before entering attachments are largely limited, which generally has often had a negative impact on our ability to fully benefit and engage at the start of clinical rotations. Consequently, we were not at all surprised by the high interest of students in the elective programme piloted by the authors, but welcomed their attempts to tackle this issue, and were further enthused by the reported positive outcomes of the elective programme on student confidence in applying clinical skills. However, as they point out, their study was limited by a small sample size and lack of generalisability. Therefore, further studies to evaluate a similar training scheme across disciplines involving larger cohorts would be of interest. While we also agree that objective assessment of student preparedness would have been of value, the positive impact on student confidence alone was a good outcome and should not be understated.

We did not agree, however, with their concern that self-selection of motivated students to such elective courses would lead to an ‘achievement gap’ over less motivated peers. Such a consideration should not preclude the need to address student preparedness, nor is there any reason to suggest that those more motivated to enrol were more likely to be high-attaining. The issues associated with transition are real, and require more concerted efforts to attenuate their effect on student experience and attainment during the clinical phases of training. In any case, effort should be made to ensure that all students are afforded the opportunity to acquire the skills and knowledge needed to succeed.

What we are concerned about is the commonly understood experience amongst medical students that those who excel most in preclinical medicine often struggle to adapt to clinical teaching methods, and decline with respect to attainment during this phase of training. In addition, there is also a perception of a polarisation between those who do well in OSCEs and those who excel most in written examinations during the clinical stage of training. While such patterns may reflect the variable individual strengths and attributes of medical students, more research should be done firstly to confirm this phenomenon, but also to understand the factors that contribute to it. Difficulty adapting to new teaching methods and the clinical environment is a known factor associated with difficulties in transition [2], and so any knowledge of student factors associated with better attainment could be clearly communicated to students prior to commencing clinics to enable them to develop effective learning strategies. Moreover, it would also enable institutions and teaching hospitals to adapt teaching methods.

Other issues associated with student transition include uncertainty with regard to intended role and responsibilities [3], adapting to a new learning environment, as well as a lack of understanding of hospital logistics [4]. All are issues which require attention, and accordingly several institutions have developed strategies to facilitate transition, including longitudinal clinical coaching programmes and transitional courses, as described in this study [5]. While the authors allude to the perceived theoretical benefits of a longitudinal approach, we feel this study demonstrated the value of intensive transitional courses in addressing these issues. Beyond early patient exposure, they offer a focused opportunity to adapt to the clinical role that is otherwise fragmented and inconsistent when offered through a longitudinal approach. As such, more effort should be exhausted to develop transitional courses as a means of promoting student preparedness, and to ensure attainment during their crucial part of training.

Finally, there is something to be said about the evidence-based approach the authors took in designing their elective programme. Highly commendable, it leaves us confident in the future with respect to medical education.
